# Supplementary material for: Sunlight-Powered Reverse Water Gas Shift Reaction Catalysed by Plasmonic Au/TiO2 Nanocatalysts: Effects of Au Particle Size on the Activity and Selectivity
Source: Nanomaterials (Basel). 2022 Nov 23;12(23):4153. doi: 10.3390/nano12234153 (PMC9738324; doi:10.3390/nano12234153)
Supplement: Supplementary file 1 [file nanomaterials-12-04153-s001.zip › nanomaterials-2001802-supplementary.pdf]

Supplementary

Sunlight-Powered Reverse Water Gas Shift Reaction Catalysed by Plasmonic Au/TiO_2 Nanocatalysts: Effects of Au Particle Size on the Activity and Selectivity

Jordi Volders [1,2,3], Ken Elen [1,2,3], Arno Raes [4,5], Rajeshreddy Ninakanti [4,5], An-Sofie Kelchtermans [1,2,3], Francesc Sastre [6], An Hardy [1,2,3], Pegie Cool [7], Sammy W. Verbruggen [4,5], Pascal Buskens [1,6,*] and Marlies K. Van Bael [1,2,3,*]

1 Design and Synthesis of Inorganic Materials (DESINe), Institute for Materials Research, Hasselt University, Agoralaan Building D, 3590 Diepenbeek, Belgium
2 Imec Vzw, Imomec Associated Laboratory, Wetenschapspark 1, 3590 Diepenbeek, Belgium
3 EnergyVille, Thor Park 8320, 3600 Genk, Belgium
4 Sustainable Energy, Air & Water Technology (DuEL), University of Antwerp, Groenenborgerlaan 171, 2020 Antwerp, Belgium
5 NANOlab Center of Excellence, University of Antwerp, Groenenborgerlaan 171, 2020 Antwerp, Belgium
6 The Netherlands Organisation for Applied Scientific Research (TNO), High Tech Campus 25, 5656 AE Eindhoven, The Netherlands
7 Laboratory of Adsorption and Catalysis, University of Antwerp, Universiteitsplein 1, 2610 Wilrijk, Belgium
* Correspondence: pascal.buskens@tno.nl (P.B.); marlies.vanbael@uhasselt.be (M.K.V.B.)

Citation: Volders, J.; Elen, K.; Raes, A.; Ninakanti, R.; Kelchtermans, A.-S.; Sastre, F.; Hardy, A.; Cool, P.; Verbruggen, S.W.; Buskens, P; Van Bael, M.K. Sunlight-Powered Reverse Water Gas Shift Reaction Catalysed by Plasmonic Au/TiO_2 Nanocatalysts: Effects of Au Particle Size on the Activity and Selectivity. *Nanomaterials* **2022**, *12*, 4153. https://doi.org/10.3390/nano12234153

Academic Editor: Andreu Cabot

Received: 15 October 2022
Accepted: 22 November 2022
Published: 23 November 2022

Publisher's Note: MDPI stays neutral with regard to jurisdictional claims in published maps and institutional affiliations.

Copyright: © 2022 by the authors. Licensee MDPI, Basel, Switzerland. This article is an open access article distributed under the terms and conditions of the Creative Commons Attribution (CC BY) license (https://creativecommons.org/licenses/by/4.0/).

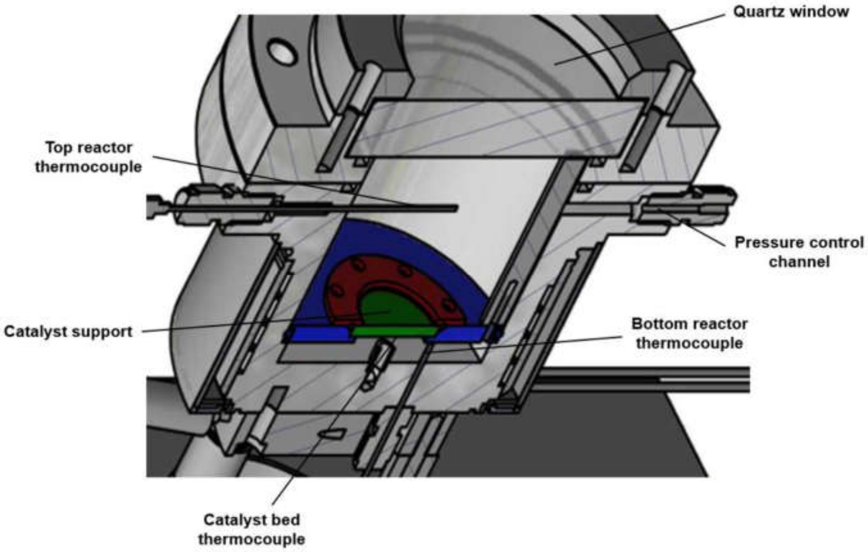

Figure S1: Schematic representation of the photocatalytic reactor vessel, including the location of the thermocouples [36].

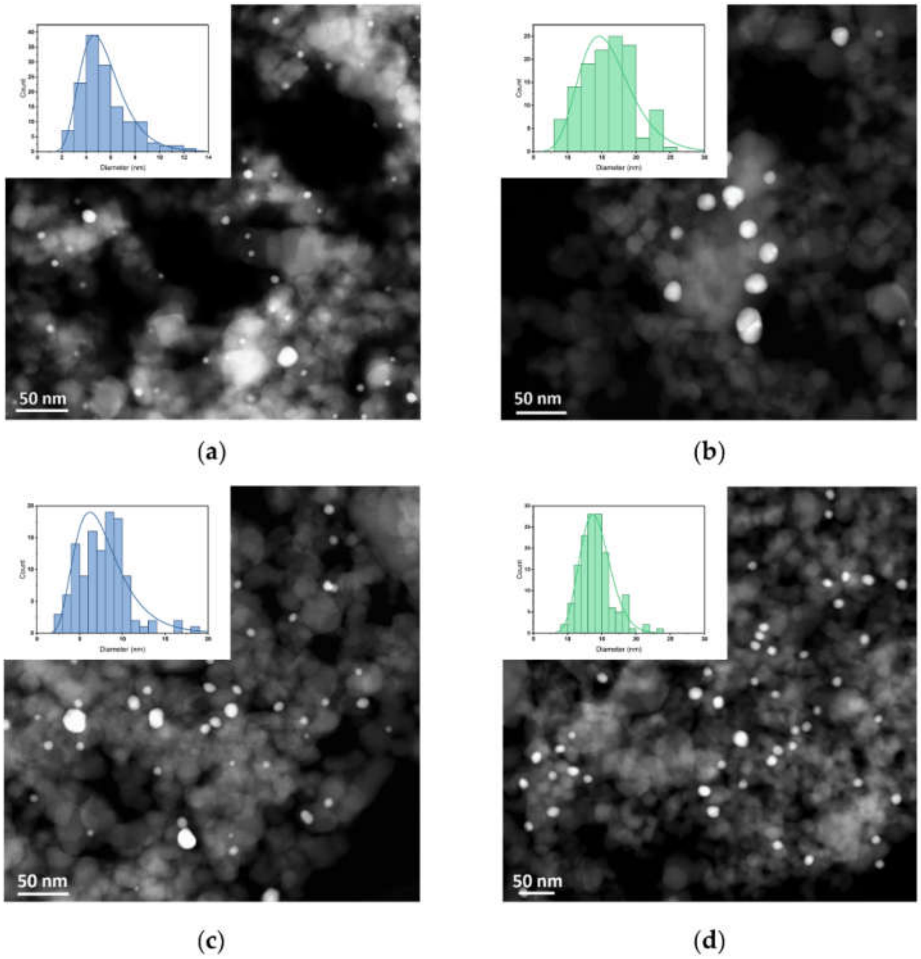

Figure S2: HAADF-STEM image of (**a**) S1; (**b**) M1; (**c**) S4; (**d**) M4 catalysts with Au nanoparticle size histogram.

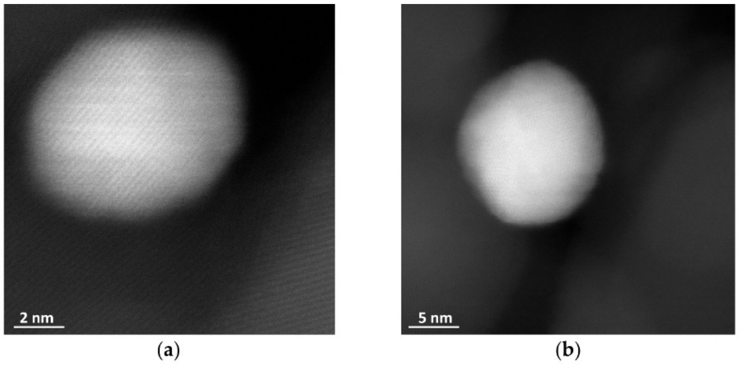

Figure S3. High-resolution scanning transmission electron microscopy images of (**a**) S2 and (**b**) M2 catalysts. No preferential orientation of the Au lattice compared to the TiO_2 lattice is found.

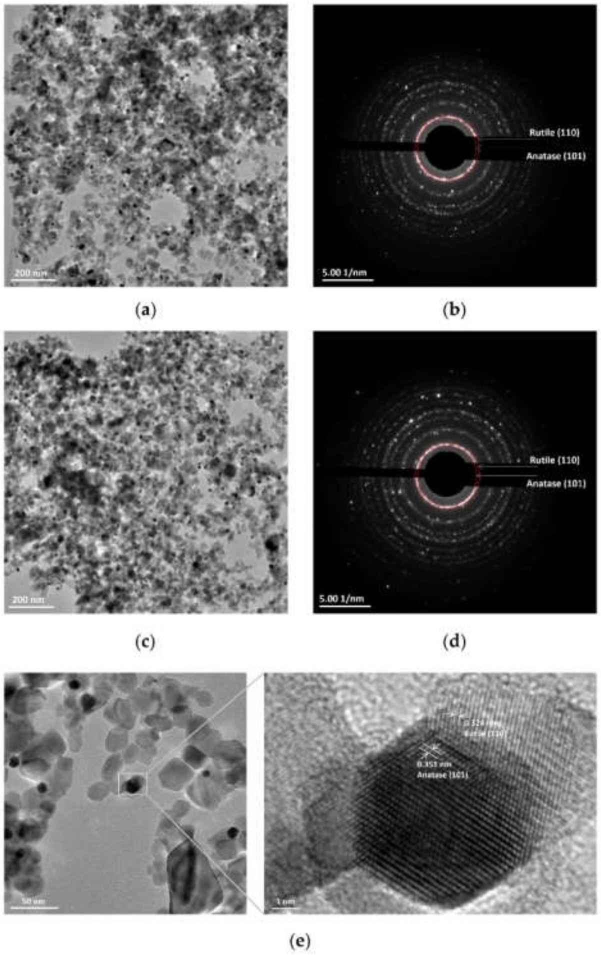

Figure S4: (a) TEM image and (b) diffraction pattern of S4 sample; (c) TEM image and (d) diffraction pattern of M4 sample; (**e**) HR-TEM with identification of anatase and rutile phases. (101) Plane of anatase and (110) plane of rutile have been marked for all images.

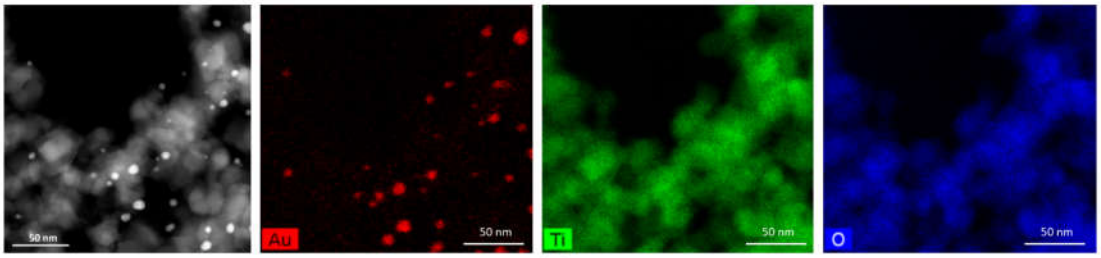

(a)

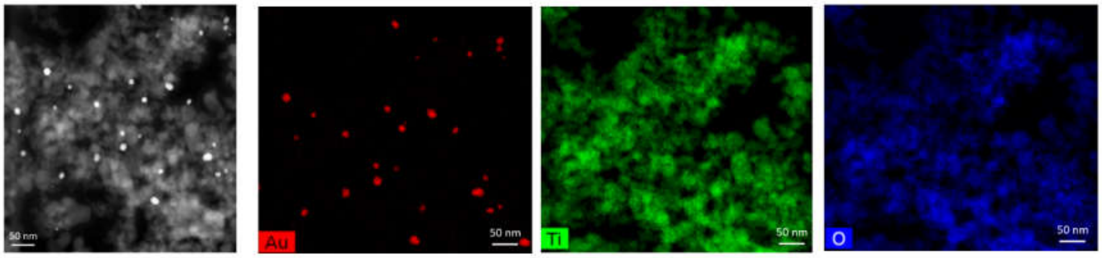

(b)

Figure S5: Energy dispersive X-ray analysis of (**a**) S2 and (**b**) M2 catalysts. Identification of Au nanoparticles on the TiO_2 support is evident.

Table S1: Elemental analysis of S4 and M4 samples using SEM-EDX, four different locations are probed, confidence intervals of 98% are reported. Only O, Ti and Au are detected. No significant difference between samples is observed.

Sample	O (wt%)	Ti (wt%)	Au (wt%)
S4	36±6%	59±6%	4±1%
M4	39±5%	59±4%	3±1%

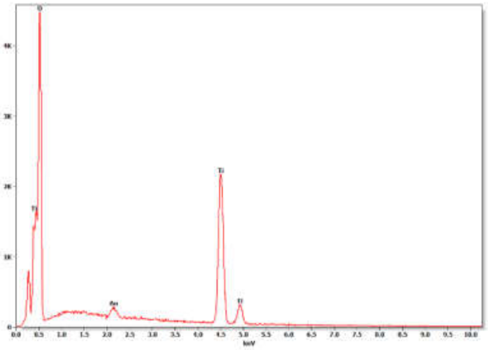

(a)

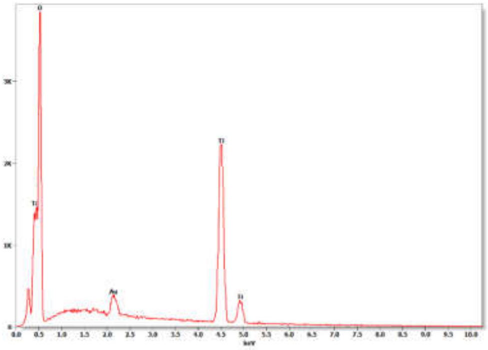

(b)

Figure S6: SEM-EDX spectrum of (**a**) M4; (**b**) S4 catalysts, O, Ti and Au peaks have been identified.

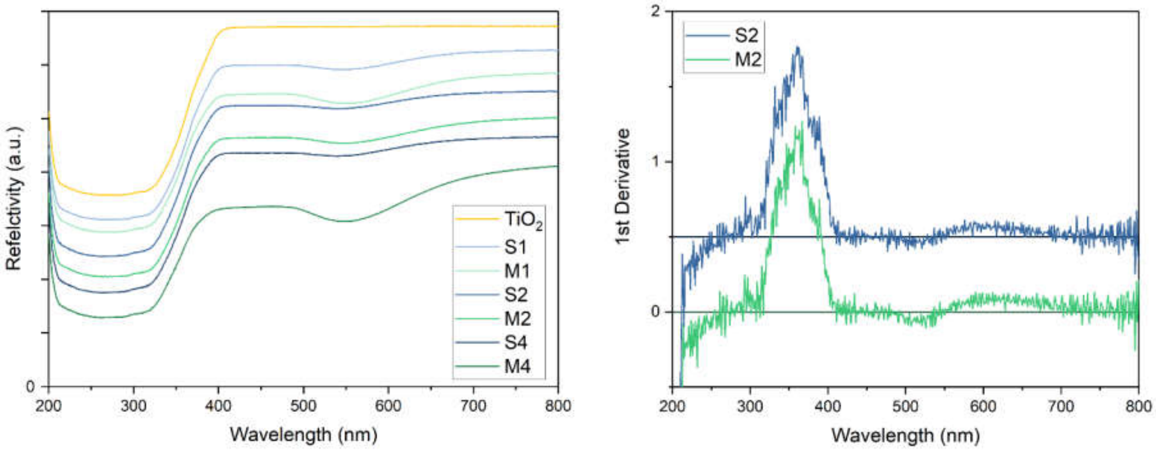

(a) (b)

Figure S7: (**a**) Diffuse reflectance UV-vis spectra for bare P25 TiO_2, S1-4 and M1-4 catalysts. Both the absorption bands of TiO_2 and plasmonic Au nanoparticles can be observed. Samples have been normalised on the intensity of the TiO_2 absorption band and stacked for visual clarity. (**b**) First derivative of the diffuse reflectance UV-vis spectra of S2 and M2 catalysts. Intersection with the reference line around 550 nm highlights the position of the plasmonic Au absorption band.

Table S2: Crystallite size determined for anatase and rutile in S4 and M4 samples using Scherrer equation. Au crystallite size could not be reliably determined due to low intensity of the diffraction peaks.

Sample	Anatase	Rutile
S4	213 Å	336 Å
M4	205 Å	299 Å

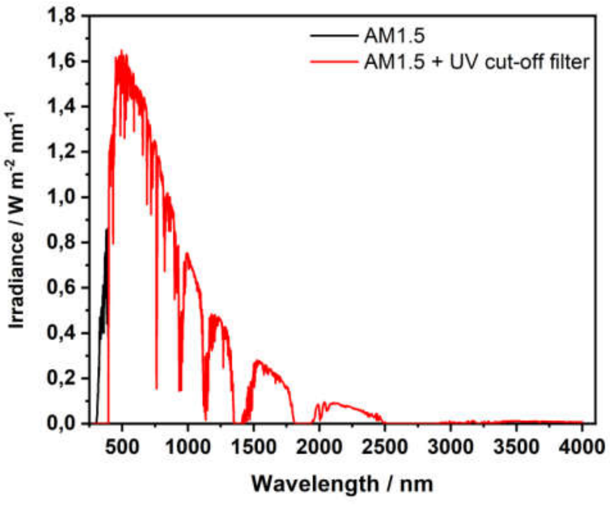

Figure S8: AM1.5 irradiance (black) of the solar simulator [36].

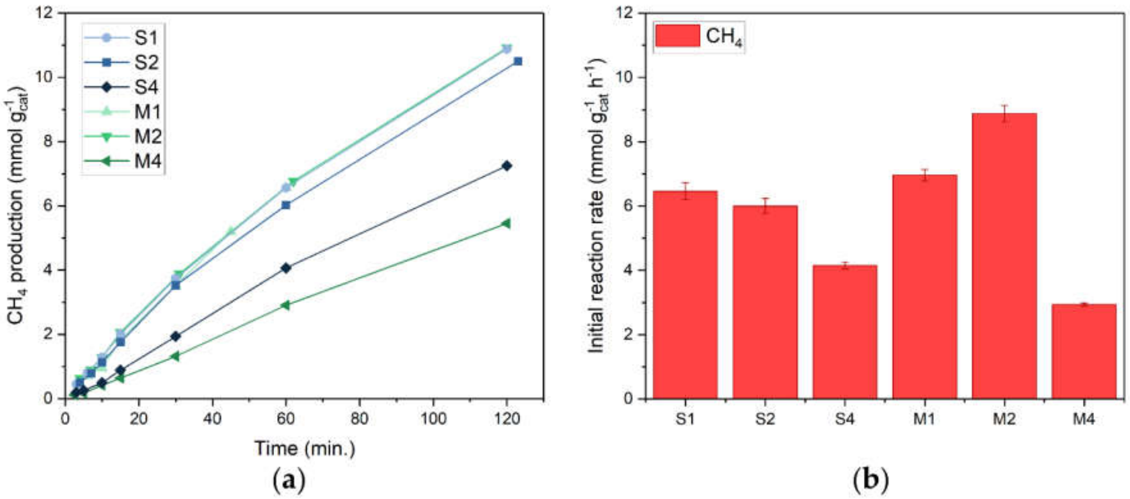

Figure S9: (**a**) CH_4 production of M1-4 and S1-4 catalysts in dark at 200°C. (**b**) Initial reaction of the M1-4 and S1-4 catalysts in dark at 200°C. Error bars represent a 98% confidence interval. Reaction conditions: mixture of CO_2:H_2:N_2 (2:2:1) at 3.5 bar pressure, 200 mg photocatalyst, dark conditions (200°C catalyst temperature).
